# Green route to synthesize Zinc Oxide Nanoparticles using leaf extracts of *Cassia fistula* and *Melia azadarach* and their antibacterial potential

**DOI:** 10.1038/s41598-020-65949-3

**Published:** 2020-06-03

**Authors:** Minha Naseer, Usman Aslam, Bushra Khalid, Bin Chen

**Affiliations:** 1 Department of Environmental Science, International Islamic University Islamabad, Islamabad, Pakistan; 2 Department of Plant Breeding and Genetics, University of Agriculture Faisalabad, Faisalabad, Pakistan; 3 Institute of Geographic Sciences and Natural Resources Research, Chinese Academy of Sciences, 11A Datun Road, Chaoyang District, Beijing, 100101 P.R. China; 4 The Abdus Salam International Centre for Theoretical Physics, Trieste, Italy; 5 Institute of Atmospheric Physics, Chinese Academy of Sciences, Beijing, 100029 China; 6 Collaborative Innovation Center on Forecast and Evaluation of Meteorological Disasters, Nanjing University of Information Science & Technology, Nanjing, 210044 China

**Keywords:** Nanoparticles, Antimicrobial resistance

## Abstract

Development of plant based nanoparticles has many advantages over conventional physico-chemical methods and has various applications in medicine and biology. In present study, zinc oxide (ZnO) nanoparticles (NPs) were synthesized using leaf extracts of two medicinal plants *Cassia fistula* and *Melia azadarach*. 0.01 M zinc acetate dihydrate was used as a precursor in leaf extracts of respective plants for NPs synthesis. The structural and optical properties of NPs were investigated by X-ray diffraction (XRD), Fourier transform infrared (FTIR) spectroscopy, scanning electron microscope (SEM), ultraviolet-visible spectrophotometer (UV-Vis) and dynamic light scattering (DLS). The antibacterial potential of ZnO NPs was examined by paper disc diffusion method against two clinical strains of *Escherichia coli* (*E. coli*) and *Staphylococcus aureus* (*S. aureus*) based on the zone of inhibition and minimal inhibitory indices (MIC). Change in color of the reaction mixture from brown to white indicated the formation of ZnO NPs. UV peaks at 320 nm and 324 nm, and XRD pattern matching that of JCPDS card for ZnO confirmed the presence of pure ZnO NPs. FTIR further confirmed the presence of bioactive functional groups involved in the reduction of bulk zinc acetate to ZnO NPs. SEM analysis displayed the shape of NPs to be spherical whereas DLS showed their size range from 3 to 68 nm. The *C. fistula* and *M. azadarach* mediated ZnO NPs showed strong antimicrobial activity against clinical pathogens compared to standard drugs, suggesting that plant based synthesis of NPs can be an excellent strategy to develop versatile and eco-friendly biomedical products.

## Introduction

Plant mediated synthesis of nanoparticles (NPs) is a revolutionary technique that has wide range of applications in agriculture, food industry and medicine. NPs synthesized via conventional methods have limited uses in clinical domain due to their toxicity. Due to the physio-chemical properties of plant based NPs, this method also offer an added advantage of increased life span of NPs that overcome the limitations of conventional chemical and physical methods of NPs synthesis^1–3^. Plants possess rich genetic variability with respect to number of biomolecules and metabolites like proteins, vitamins, coenzymes based intermediates, phenols, flavonoids and carbohydrates. These plant metabolites contain hydroxyl, carbonyl, and amine functional groups that react with metal ions and reduce their size into nano range. More specifically, flavonoids contain several functional groups and it is believed that -OH group of flavonoids is mainly considered responsible for the reduction of metal ions into NPs^4^. These molecules not only help in bioreduction of the ions to the nano scale size, but they also play a pivotal role in the capping of the nanoparticles which is important for stability and biocompatibility^5^. Reducing agents such as phenolic compounds, sterols and alkaloids can reduce metal ions into NPs in a single reaction^6^.

The type and nature of the metal used for NPs biosynthesis mainly determines the NPs end use industry. Several metals such as silver (Ag), copper (Cu), gold (Au) and many others have been widely used for the biosynthesis of NPs using plant extracts of various plant species^7–9^. However their higher toxicity to animals and humans pose a serious limitation for use in medical industry. ZnO is an inorganic compound which occurs rarely in nature. It is generally found in crystalline form. Naturally occurring ZnO has manganese impurities that give it a typical red or orange color appearance^10^. When purified, ZnO appears as white crystalline powder which is nearly insoluble in water. Due to their low toxicity and size dependent properties, ZnO NPs have been widely used for various applications in textiles, cosmetics, diagnostics and even in micro-electronics. Because ZnO is generally recognized as safe (GRAS) and exhibits antimicrobial properties, ZnO NPs hold greater potential to treat infectious diseases in humans and animals^11^.

ZnO has been found to be potentially useful and efficient than other metals for biosynthesis of NPs for clinical purposes. Several studies have demonstrated the synthesis of ZnO NPs using different plant extracts. For example, flower extract of the medicinal plant *Cassia auriculata*^12^ and leaf extract of *Hibiscus rosasinensi*^13^ were used as reducing agents for zinc nitrate to synthesize ZnO NPs.

Plant type or source species from which plant extract used for NPs synthesis also affects the size of NPs. For example, when *Olea europea* leaf extract was used to synthesize ZnO nano sheets, it ranged from 18–30 nm in size^14^. However, when *Aloe barbadensis*^15^ and *Ocimum tenuiflorum*^11^ were used as reducing agent for the green synthesis of ZnO nanoparticles, the average nanoparticle sizes were 25–40 nm and 13.86 nm respectively. Recently various reports have also demonstrated the antimicrobial activity of ZnO NPs. For example, ZnO NPs synthesized by using leaf extracts of *Passiflora caerulea*, *Scadoxus multiflorus* and *Camellia sinensis* showed strong antimicrobial efficacy against *Klebsiella pneumonia*, *Aspergillus* spp., and *Staphylococcus aureus* and *Pseudomonas aeruginosa* respectively^16–18^, suggesting that medicinal plant extract mediated synthesis of ZnO NPs can be very useful for medical industry.

*Cassia fistula* commonly known as Golden Shower or Amaltas is a deciduous tree with medicinal importance, native to Pakistan and India and found as an exotic species in Egypt, Australia, Ghana, Mexico, and Zimbabwe. It belongs to the family *Fabaceae*. It produces shiny green leaves which are about 30–40 cm long, pinnate in shape and arranged in alternate fashion on the terminal branches^19^. Leaves of *C. fistula* contain a wide variety of antioxidants for example; terpenoids, flavonoids, alkaloids, phenolic compounds, tannins, saponins, anthocyanosides, carbohydrates, proteins, steroids, cardiac glycosides and phlobatannins^20^.

Similarly, *Melia azadarach* commonly known as Cape Lilac and locally as Bakain belongs to the family *Meliaceae*. It is native to Southeast Asia and found naturally in most of the tropical and subtropical countries. This plant is locally famous for its anti-microbial, anti-inflammatory and anti-cancer activities and often used to treat stomach pains and parasitic infections. It produces dense array of dark green leaves which are short stalked and arranged in alternate pattern on terminal branches. Fruits are yellow colored, smooth and fleshy berries. *M. azadarach* is naturally enriched in phytochemicals. It is endowed with alkaloids, sterols, glycosides, flavonoids, limonoids, fixed oil and fats, phenolic compounds, tannins, saponins, gum and mucilages, triterpenes, azadirachitin, nimbin, melianoninol, melianol, meliandiol, vanillin, meliacin, quercertin and rutin^21^. Due to the presence of diverse array of these phytochemicals and medicinal properties, *C. fistula* and *M. azedarach* hold greater potential for efficient biosynthesis of NPs that can be useful to treat clinical pathogens.

Here, we report a simple and eco-friendly method of ZnO NPs synthesis from the plant extracts of *C. fistula* and *M. azedarach* as reducing agents and zinc acetate as precursor for their comparative analysis of antimicrobial potential. This research will increase the potential of usage of plant based NPs in biomedical industry.

## Results and Discussion

### Optical analysis of ZnO NPs formation

**Figure 1:**
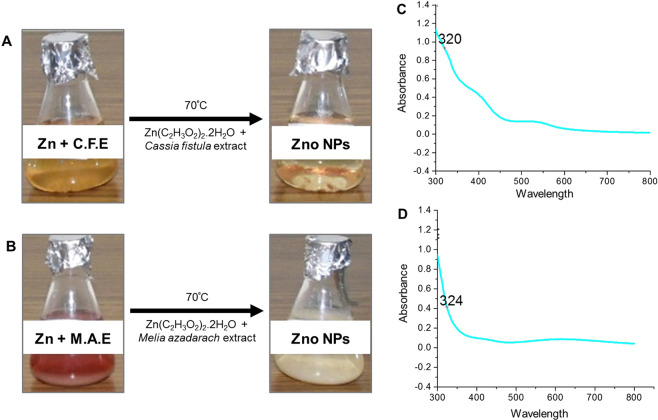
Optical analysis of ZnO NPs. (**A,B**) Color changes indicating formation of ZnO NPs. A) *Cassia fistula* mediated ZnO NPs. (**B**) *Melia azadarach* mediated ZnO NPs. (**C,D**) UV-visible absorption spectrum confirming presence of ZnO NPs. (**A**) *Cassia fistula* mediated ZnO NPs. (**B**) *Melia azadarach* mediated ZnO NPs.

Adding zinc acetate dihydrate in leaf extracts of *C. fistula* and *M. azedarach* leads to physio-chemical changes in the aqueous solution. The most prominent of which is change in the colour of the reaction mixture that can be observed within few minutes. This was considered as an initial signature to formation of NPs. In present study, change of color from yellow to light brown and red to off-white indicated the formation of ZnO NPs in leaf extracts of *C. fistula* and *M. azedarach*, respectively. Flavonoides and phenolic compounds are thought to be responsible for Zn ions to ZnO NPs. In a period of few hours, the colour of the solution stopped changing further suggesting the complete bioreduction of ZnO salt into NPs. A clear illustration of change in color of the reaction mixtures due to formation of ZnO NPs has been shown in Fig. 1(A,B). These results were consistent with the previous reports of color changes in plant based synthesis of ZnO NPs^22^. Temperature is considered an important contributing factor in synthesis of good sized nanoparticles. It is also well established that higher the temperature of reaction process of NPs synthesis, the smaller the size of the NPs^23,24^. Therefore, we use a relatively higher temperature of 70 °C for incubating the reactants that leads to the production of very small sized ZnO NPs.

The synthesis of ZnO NPs was further examined by UV spectrophotometry. Figure 1(C,D) shows the UV peaks recorded by the spectrophotometer. The maximum absorption peak for ZnO NPs synthesized via *C. fistula* was recorded at 320 nm and with that of *M. azadarach* at 324 nm that further verified the formation of ZnO NPs. Firstly, these results satisfy standard ZnO absorption pattern because all oxide materials have wide band gaps and tend to have shorter wavelengths. Moreover, if the material is of nanoscale, it tends to have further shorter wavelengths. This notion support the results observed for ZnO NPs here^25^.

### Surface morphology of ZnO NPs

**Figure 2:**
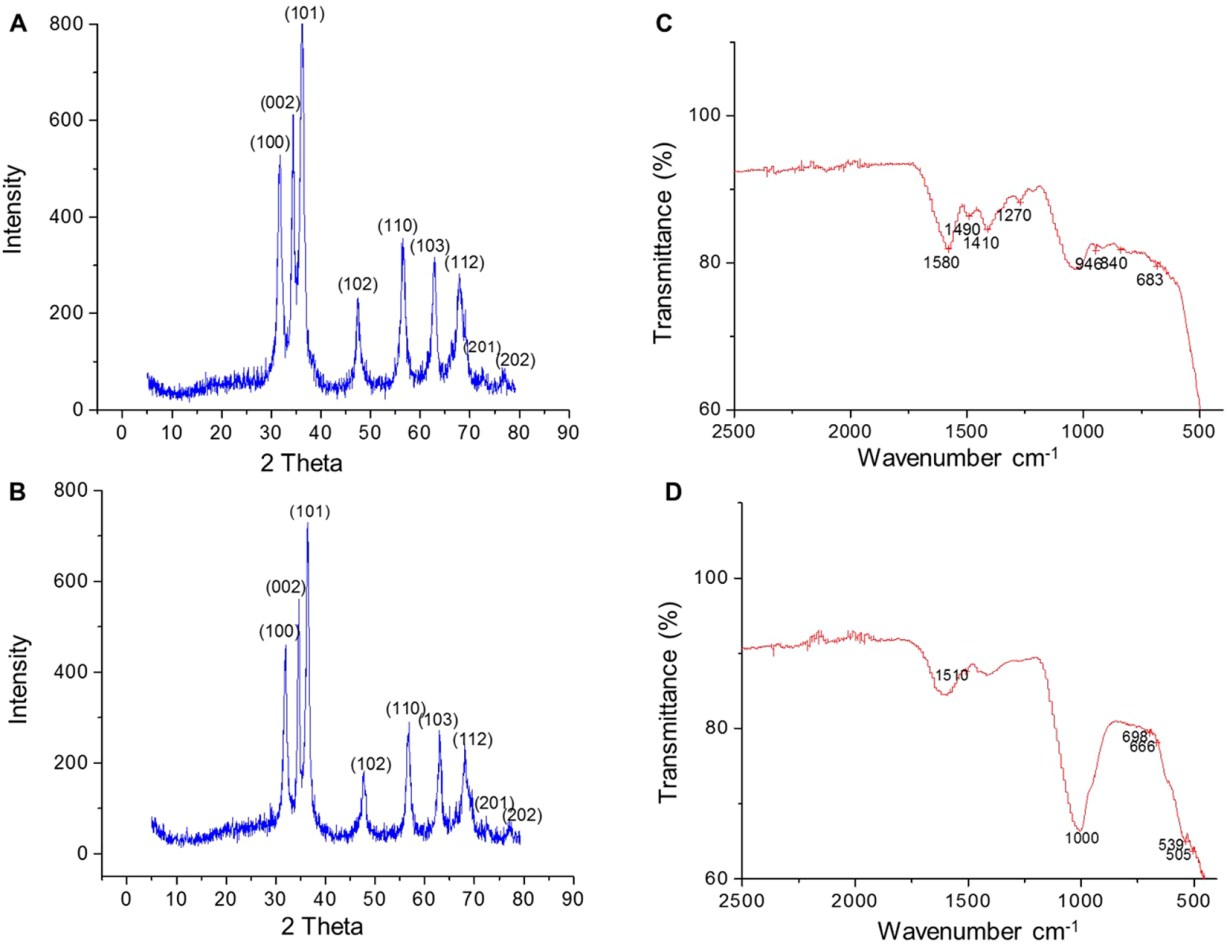
(**A,B**) XRD pattern indicating presence of ZnO peaks. (**A**) *Cassia fistula* mediated ZnO NPs. (**B**) *Melia azadarach* mediated ZnO NPs. (**C,D**) FTIR pattern indicating the functional groups involved in ZnO NPs synthesis. (**C**) *Cassia fistula* mediated ZnO NPs. (**D**) *Melia azadarach* mediated ZnO NPs.

The presence of nanoparticles and examination of their structural properties were confirmed by X-ray diffractrometer. *C. fistula* and *M. azedarach* associated ZnO NPs showed peaks with 2θ values identified at 31.841°, 34.507°, 36.324°, 47.592°, 56.634°, 66.426°, 67.983°, 69.091°, and 76.987° which are indexed as (100), (002), (101), (102), (110), (103), (112), (201) and (202) planes (Fig. 2A,B). These peaks were in accordance with those of data card (JCPDS-36-1451). Average crystal size calculated using the Scherrer’s equation ($Dp\,of\,ZnO\,NPs=(0.9(1.5406)/0.63(\cos \,36)$ came out to be around 2.72 nm for both *C. fistula* and *M. azedarach* associated ZnO NPs that is comparable with the size of good quality NPs in existing reports^26^.

To identify the functional groups associated with the ZnO NPs formation, FTIR spectrometry was performed. Spectral peaks at 683–500 cm^−1^ and 698–505 cm^−1^ proposed the formation of ZnO nanoparticles in *C. fistula* and *M. azedarach* extracts, respectively (Fig. 2C,D). Absence of peaks in the region of 3500 and 2500 cm^−1^ indicated no characteristic OH and N-H stretching of aldehydes. The bands at 1600–1510 cm^−1^ correspond to amide I and amide II regions arising due to carbonyl stretching in proteins and that of 1400 to 1000 cm^−1^ correspond to methylene from the proteins in the solution and C-N stretching vibrations of amine. Peaks from 1460–1410 cm^−1^ suggested C-C stretching vibration of alcohol, carboxylic acid, ether and ester and bands at 946–769 cm^−1^ demonstrated presence of carboxylic acid and aromatic C-H bending. Although, many changes were not observed at these frequencies but all peaks showed a shift to lower frequency and a decrease in intensity on binding with the nanoparticles. This trend of free carbonyl and NH_2_ groups from proteins and amino acid residues indicates that they have ability to bind to a metal and that the proteins could possibly form a layer around the metal for preventing agglomeration and thereby stabilizing the nanoparticles. It is revealed from the FTIR spectra that in fact, the protein molecules present in the leaf extract possibly cause the reduction of metal ions which is in agreement with the previous reports^27^. These findings suggest that not only the OH group of flavonoids but also the protein molecules and their functional groups play important role in bioreduction of salts and capping of NPs.

**Figure 3:**
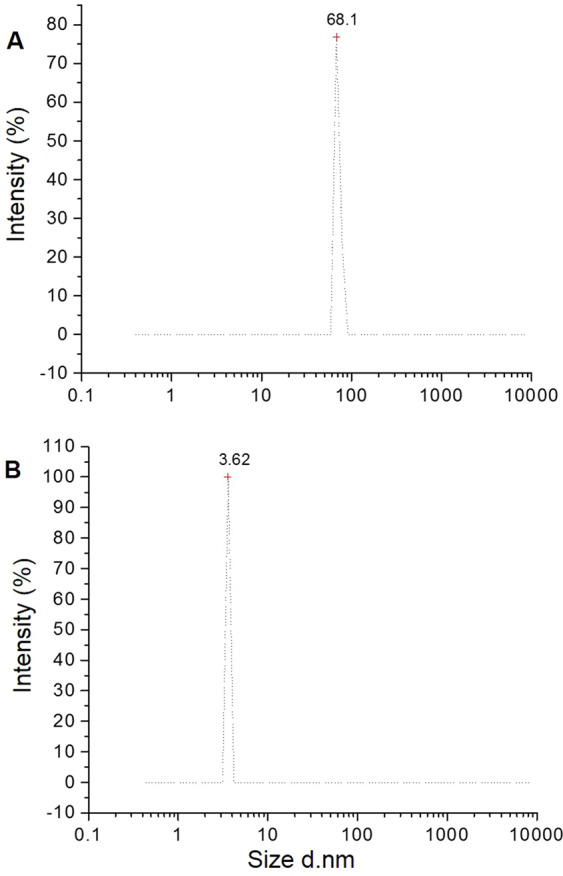
DLS indicating average size of ZnO NPs. (**A**) *Cassia fistula* mediated ZnO NPs. (**B**) *Melia azadarach* mediated ZnO NPs.

Dynamic Light Scattering (DLS) measurements showed the average diameters of *C. fistula* and *M. azadarach* mediated ZnO NPs (Fig. 3). Average diameters of ZnO NPs synthesized from *C. fistula* and *M. azadarach* were 68.1 nm and 3.62 nm, respectively. The results demonstrated that the particles synthesized were ultrafine i.e. less than 100 nm in diameter. It clearly depicts that *M. azadarach* extract was more efficient than *C. fistula* for synthesizing smaller NPs. It may be attributed to the presence of more variety of phytochemicals in *M. azadarach* when compared to *C. fistula*. As it has already been mentioned in the introduction section that *M. azadarech* possesses complete set of phytochemicals that can be the reason behind higher efficacy of this plant as a reducing agent when compared to *C. fistula*. In addition, DLS analysis demonstrated that the NPs formed had fairly well-defined dimensions^28^. Smaller the size of the NPs, higher the surface area, thus higher the antimicrobial activity. Generally, bacterial cellular membranes have nanometer size. If the nanoparticles are smaller in size than cell membrane pores, there is more possibility of crossing the cell membrane barrier and thus inhibiting the bacterial growth^29^.

**Figure 4:**
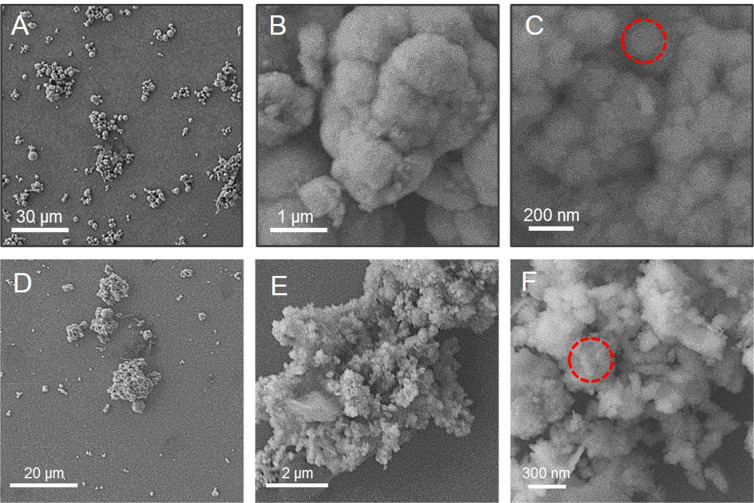
SEM images of ZnO particles showing their morphology at three different resolutions. (**A–C**): Scanning Electron Micrographs of *Cassia fistula* mediated Zno NPs. (**A**) SEM of Zno NPs captured at 500× magnification. (**B**) SEM of Zno NPs captured at 16,000× magnification. (**C**) SEM of Zno NPs captured at 65,000× magnification. (**D–F**): Scanning Electron Micrographs of *Melia azadarach* mediated Zno NPs. (**D**) SEM of Zno NPs captured at 800× magnification. (**E**) SEM of Zno NPs captured at 8,000× magnification. (**F**) SEM of Zno NPs captured at 30,000× magnification. Red dotted circles in (**C,F**) indicate the NPs circumference.

Figure 4 shows Scanning Electron Microscopy (SEM) images of ZnO NPs synthesized from leaf extracts of *C. fistula* and *M. azadarach*. The images were recorded at magnification of 10 µm, 1 µm and 100 nm. Topographical view shows that nanoparticles are more or less spherical in nature, clustered together and surface of the aggregates seems to be rough^30^. SEM images also revealed that NPs derived from both plants are entirely pure and it can be concluded that both the plants have tremendous capability to synthesize ZnO NPs. Shape of NPs plays very crucial role in the effectivity against pathogens. Because spherical NPs tend to be very potent during antibacterial activity owing to their ability to easily penetrate into the cell wall of pathogens^31^, therefore, ZnO NPs syntheized from these two plant species can be of great importance in treating clinical pathogens.

### Antibacterial activity of ZnO NPs

**Figure 5:**
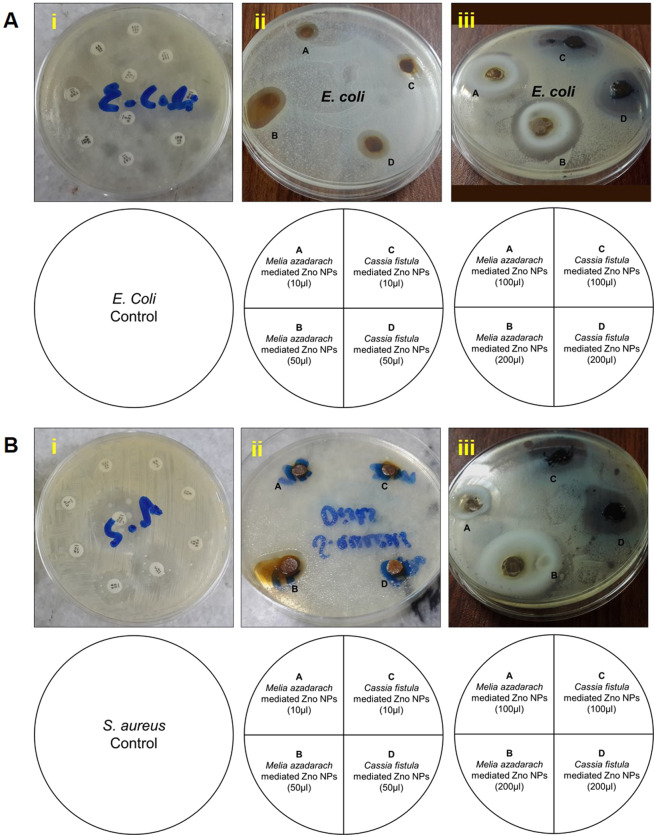
Resistance level of two clinical pathogens against (i) standard drugs, (ii) ZnO NPs 10 µL, 50 µL and (iii) ZnO NPs 100 µL, 200 µL. (**A**) Inhibition zones of ZnO NPs against *E. coli* growth. (**B**) Inhibition zones of ZnO NPs against *S. aureus* growth. Lower panel in both part A and B illustrates the labelling of petri plates.

The bactericidal activities of *C. fistula* and *M. azadarach* mediated ZnO NPs were tested against two main clinical pathogens; a (Gram-negative pathogen) *E. coli* and b (Gram-positive pathogen) *S. aureus*. Figure 5 illustrates zones of inhibition of *E. coli* and *S. aureus* against standard drugs and biosynthesed ZnO NPS at concentrations ranging from 50 µg/mL (10 µL) to 1000 µg/mL (200 µL). The mean values of zone of inhibition (mm) of three replicates are presented in (Table 1). Comparison between standard antibiotics and biosynthesed NPs showed strong antibacterial effect of NPs as compared to standard drugs (Table 2). In *E. coli*, zone of inhibition of standard drugs ranged from 15–20 mm while that of ZnO NPs was 16–40 mm. *S. aureus* was resistant to a variety of standard drugs and zone of inhibition for rest of the standard drugs was ranged from 4–13 mm while that of ZnO NPs was 14–37 mm in range. (Table 3) shows zones of inhibition of various standard drugs and standard drug potency according to WHO standards.

**Table 1:** Antibacterial Activity of ZnO NPs.

Sr No.	Bacterial Species	Zone of Inhibition (mm)			
		*Cassia fistula* mediated ZnO NPs		*Melia azadarach* mediated ZnO NPs	
		10 µl	200 µl	10 µl	200 µl
1.	*E. coli*	21 ± 0.68	44 ± 3.00	20 ± 0.56	40 ± 0.48
2.	*S. aureus*	14 ± 0.54	32 ± 2.30	21 ± 0.68	38 ± 0.55

**Table 2:** Comparison among inhibition zones of nanoparticles and antibiotics against clinical strains.

Sr No.	Clinical Strains	Zone of inhibition in mm					
		Antibiotics	Zone	*Cassia fistula*		*Melia azadarach*	
				ZnO NPs		ZnO NPs	
				10 µl	200 µl	10 µl	200 µl
1.	*E. coli*	Ceftazidime	20 ± 0.04	21 ± 0.068	44 ± 0.300	20 ± 0.56	40 ± 0.48
		Imipenem	22 ± 0.02				
		Cefoperazone	19 ± 0.21				
		Amoxicillin	18 ± 0.09				
		Amikacin	15 ± 0.085				
		Ceftriaxone	16 ± 0.16				
		Tobramycin	17 ± 0.17				
		Levoflaxin	18 ± 0.11				
		Tazobactum	18 ± 0.07				
2.	*S. aureus*	Erythromycin	R	14 ± 0.054	32 ± 0.230	21 ± 0.68	38 ± 0.55
		Gentamycin	4 ± 0.45				
		Vancomycin	5 ± 0.32				
		Chloramphenicol	12 ± 0.05				
		Cefoxitin	R				
		Clindamycin	R				
		Co trimonazole	R				
		Ciprofloxacin	R				

Mean values of three replicates ± standard deviation. R stands for resistant.

**Table 3:** Inhibition zones and potency of standard drugs used according to WHO standards.

Standard Drug	Abbreviations	Zone of inhibition in mm (WHO standard)	Drug Potency (µg)
Amikacin	(AK)	>17	30
Ceftriaxone	(CRO)	>26	30
Amoxicillin	(AMC)	>18	30
Ceftazidime	(CAZ)	>21	30
Cefixime	(CFM)	>19	10
Imipenem	(IPM)	>23	10
Cefoperazone	(SCF)	>21	105
Tazobactum	(TZP)	>18	110
Tobramycin	(TOB)	>15	10
Levoflaxin	(LEV)	>19	5
Cefoxitin	(FOX)	>23	30
Erythromycin	(E)	>23	15
Clindamycin	(DA)	>21	2
Cotrimonazole	(SXT)	>21	25
Ciprofloxacin	(CIP)	>21	5
Gentamycin	(CN)	>15	10
Vancomycin	(VA)	>16	30
Chloramphenicol	(C)	>18	30
Lanzolid	(LZP)	>21	30
Tetracycline	(TCN)	>19	30

Both the *E. coli* and *S. aureus* showed minimum inhibitory concentration (MIC) at 10 µL for the synthesized ZnO NPs. Furthermore, as the concentration of NPs increased so did the zone of inhibition. It is evident from the recordeded images and statistical data that zone of inhibition of *C. fistula* mediated ZnO NPs was more significant against *E. coli* (∼44 mm) as compared to *S. aureus* (Fig. 5, Table 2). The mild inhibitory effect of *C. fistula* mediated ZnO NPs on *S. aureus* when compared to *E. coli* can be attributed to the differences in membrane strutures of Gram-positive and Gram-negative bacteria. The most disntinctive feature of Gram-positive bacterium is the thickness of cell wall due to the prescence of peptidoglycan layer. It has also been reported that ZnO NPs may damge bacterial cell membrane resulting lysis of intracellular contents and ultimately proved to be lethal for the bacterial cell^32^. Lower efficacy of *C. fistula* mediated ZnO NPs against *S. aureus* compared to the Gram-negative species might be due to the resistance of cell wall in Gram-positve species^33^. By contrast, the zone of inhibition of *M. azadarach* mediated ZnO NPs was compareable against both the pathogens. However, it is important to note that the zone of inhibition of *M. azadarch* mediated ZnO NPs was significantly greater in comparison to *C. fistula* mediated ZnO NPs against *S. aureus* (Fig. 5, Table 2). These results suggest that the use of *M. azadarch* mediated synthesis of ZnO NPs can be more efficient against Gram-positive pathogens like *S. aureus*. This might be due to the presence of higher number of phenolic compounds and rare secondary metabolites such as nimbinene, meliacin, quercertin and rutin in *M. azadarch*.

**Figure 6:**
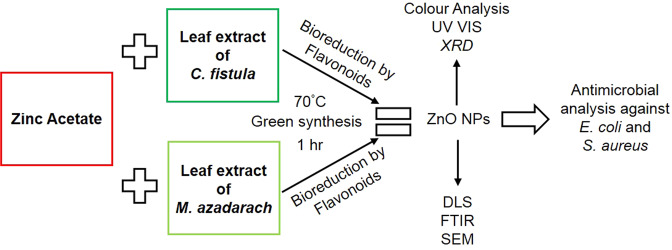
Schematic model of ZnO NP synthesis from the leaf extracts of *Cassia fistula* and *Melia azedarach* and their antibacterial activity analysis.

As a schematic layout of this whole study, a model has been given in Fig. 6 that shows the graphical representation of the synthesis of ZnO NPs using leaf extarcts of *C. fistula* and *M. azadarach* as reducing agents and zinc acetate as a precursor salt.

## Conclusion

Leaf extracts of *C. fistula* and *M. azadarach* showed excellent potential as reducing agents in the formation of NPs. Structural and optical studies conducted using UV, FTIR, XRD, DLS and SEM analysis confirmed the formation of efficient ZnO NPs. Antibacterial analysis revealed that ZnO NPs synthesized from leaf extracts exhibited significant capability of inhibition against the clinical pathogens when compared to traditional drugs. Moreover, some plant extratcs are more effective than that of others in synthesizing NPs and biological activities due to their diverse biochemical compositions. In conclusion, synthesis of NPs using extratcs of medicinal plants can have useful medicinal applciations in treatment of numerous human infectious pathogens. However, further studies will be required to validate the efficacy of these NPs in medical applications and their capacity to overcome the risks associated with conventional drugs.

## Methods

### Synthesis of Nanoparticles

All the glassware were autoclaved before use. To prepare leaf extract, fresh leaves of *C. fistula* and *M. azadarach* were thoroughly washed with tap water followed by distilled water (d.H_2_O) to remove any contamination. The leaves were air dried for a week at room temperature (∼37°C). About 5 g of leaves from each of *C. fistula* and *M. azadarach* were ground to fine powder with the help of pestle and mortar. This powder was mixed in 500 mL of d.H_2_O and then heated at 70°C for 30 minutes. The mixture was filtered first by muslin cloth and then using Whatman filter paper No.1. As a result, pale yellow and red colored solutions were obtained as leaf extracts of *C. fistula* and *M. azadarach* respectively which were stored at 4 °C.

0.01 M zinc acetate dihydrate (Zn (C_2_H_3_O_2_)_2_.2H_2_O) solution was prepared in d.H_2_O. For synthesis of ZnO nanoparticles, 95 mL of 0.01 M zinc acetate dihydrate (Zn (C_2_H_3_O_2_)_2_.2H_2_O) solution was mixed separately with 5 mL plant extract of each of *C. fistula* and *M. azadarach* in individual 250 mL flasks. These mixtures were incubated at 70°C for 1 hour with continuous shaking at 150 rpm. This led to the settlement of bio-reduced salt at the bottom of the flask which appeared as white precipitate. The supernatant was decanted and powdery precipitate was transferred to 1.5 mL centrifuge tubes. Both the samples were subjected to washing with d.H_2_O by centrifugation at 3000 rpm for 30 minutes. Washing step was repeated thrice to ensure removal of impurities^22^.

### Characterization of NPs

*Optical Spectroscopy*. To measure the optical parameters, ZnO synthesized nanoparticles were dispersed in d.H_2_O. The absorption spectrum of synthesized NPs was measured using UV–VIS-NIR spectrophotometer (UV-1601, Shimadzu, Japan) in wavelength range between 200–800 nm. The d.H_2_O was used as a reference. Energy gap or band gap was calculated using the following equation

$${\rm{Eg}}=1239.83/\lambda \,{\rm{nm}}$$

where Eg is the bulk band expressed in eV. Lambda (𝜆) is peak absorbance wavelength in nm. Therefore, the energy gap for ZnO ranges from 4.27–3.87 eV^34^.

*FTIR Analysis*. The surface chemistry of NPs was analyzed by FTIR spectroscopy. The functional groups attached to the surface of NPs were detected in the range of 4000–400 nm. The samples were prepared by dispersing the ZnO NPs uniformly in a matrix of dry KBr which was then compressed to form a transparent disc. KBr pellet was used as a standard^35^.

*XRD Analysis*. X-ray diffractrometer (PAN analytical X-Pert PRO) was used to study the surface morphology, size and crystalline nature of ZnO NPs. The diffraction pattern was obtained using CuKα radiation with wavelength of λ = 1.541 A°. A thin film of the sample was made by putting a small amount of sample on a glass plate for XRD studies. The scanning was done in 2θ value range of 4° to 80° at 0.02 min^−1^ and 1 second time constant. The instrument was operated at a current of 30 mA and voltage of 40 kV. Scherrer’s equation was used to calculate the average grain size of synthesized NPs which is as under

1 $${\rm{Dp}}=0.9\lambda /\beta \,\mathrm{Cos}\,{\rm{\theta }}$$

where D represents the crystallite size, λ stands for the wavelength (1.5406 Å for Cu Kα), β symbolizes the full-width at half-maximum (FWHM) of main intensity peak after subtraction of the equipment broadening and θ is used as a diffraction angle in radians.

*DLS Analysis*. The particle size distribution of the samples was obtained through Particle Size Analyzer (Zetasizer Ver. 7.11 Malvern). The liquid samples of ZnO NPs was diluted ten times using Milli-Q water, centrifuged and then transferred to cuvette for analysis. The zeta potential of ZnO NPS was determined in water as dispersant.

*SEM Imaging*. The samples of ZnO NPs were dispersed in methanol (evaporating solvent) at a concentration of 1 mg/20 mL. A single drop of aqueous solution of ZnO NPs was placed on the carbon coated grid to prepare a thin film. Extra solution was removed with the help of blotting paper and the grid was allowed to dry under mercury lamp for around five minutes. The morphological measurements of the ZnO NPs samples were recorded with field emission scanning electron microscope (JEOL, Model: JSM-7600F) in the range of 0.1 nm to 10,000 nm. The data collected from all techniques was analyzed in Origin software version 9.1.

### Antimicrobial analysis

To check the bactericidal potential of the NPs, pure cultures of *Escherichia coli* (EPEC-A (P16), and *Staphylococcus aureus* [(MRSA belonging to clonal complex 8 (CC8) and sequence type 239 (ST239)] were obtained from the Department of Microbiology, Pakistan Institute of Medical Sciences (PIMS), Islamabad. Disc diffusion method was used to carry out the antibacterial assay of NPs on Muller Hinton Agar (MHA) medium containing petri plates. Contamination test was carried out by incubating the plates over night at room temperature. After confirmation of no contamination, bacterial cultures were streaked on to these MHA plates.

Stock solution of NPs was prepared in d.H_2_O at a concentration of 5 mg/mL. Further, four working dilutions i.e. 50 µg/mL (10 µL), 250 µg/mL (50 µL), 500 µg/mL (100 µL) and 1000 µg/mL (200 µL) were made to find out minimum inhibitory concentration (MIC). The Minimum Inhibitory Concentration (MIC) of the ZnO NPs was determined based on batch cultures containing varying concentrations of ZnO NPs in suspension (10–200 µg/mL). Bacterial concentrations were determined by measuring optical density (OD) at 600 nm.

To examine the bactericidal effect of NPs on clinical strains, approximately 10^8^ CFU of each strain was cultured on nutrient agar plates. Following disc diffusion method, the sterile discs were dipped in ZnO nanoparticles solution at varying concentrations from 50 µg/mL to 1000 µg/mL. Discs were placed onto the MHA plates and incubated at 37 °C. Control samples were prepared by placing standard medicine discs onto MHA plates containing bacterial isolates. Standard medicines used for *E. coli* were Ceftazidime, Imipenem, Cefoperazone, Amoxicillin, and Cefixime, whereas, Erythromycin, Gentamycin, Vancomycin, Chloramphenicol, Lanzolid were used for *S. aureus*. Mean values of inhibitory zone diameter were recorded in three experimental repeats. The average values of inhibition zones were calculated as Mean ± Standard Deviations. The data was statistically analyzed using Origin software version 9.1^36^.
